# Unraveling the Effect of Immunogenicity on the PK/PD, Efficacy, and Safety of Therapeutic Proteins

**DOI:** 10.1155/2016/2342187

**Published:** 2016-08-08

**Authors:** Alison Smith, Hugh Manoli, Stacey Jaw, Kimberley Frutoz, Alan L. Epstein, Leslie A. Khawli, Frank-Peter Theil

**Affiliations:** ^1^Department of Pathology, Keck School of Medicine of University of Southern California, Los Angeles, CA 90089, USA; ^2^Nonclinical Development, UCB Biopharma SPRL, 1420 Braine-l'Alleud, Belgium

## Abstract

Biologics have emerged as a powerful and diverse class of molecular and cell-based therapies that are capable of replacing enzymes, editing genomes, targeting tumors, and more. As this complex array of tools arises a distinct set of challenges is rarely encountered in the development of small molecule therapies. Biotherapeutics tend to be big, bulky, polar molecules comprised of protein and/or nucleic acids. Compared to their small molecule counterparts, they are fragile, labile, and heterogeneous. Their biodistribution is often limited by hydrophobic barriers which often restrict their administration to either intravenous or subcutaneous entry routes. Additionally, their potential for immunogenicity has proven to be a challenge to developing safe and reliably efficacious drugs. Our discussion will emphasize immunogenicity in the context of therapeutic proteins, a well-known class of biologics. We set out to describe what is known and unknown about the mechanisms underlying the interplay between antigenicity and immune response and their effect on the safety, efficacy, pharmacokinetics, and pharmacodynamics of these therapeutic agents.

## 1. Introduction

Biologics are some of the most promising innovations in modern drug therapy and represent a diverse array of molecular tools ranging in complexity and size. These biologically derived therapeutic agents are engineered by exploiting molecular and cellular machinery already found in nature. They are capable of performing complex and precise functions; their medicinal applications are seemingly endless. They greatly differ from the more traditional concept of a* drug*: the small molecule. Small molecules are typically orally administered, lipophilic, organic compounds that are low in molecular weight (<900 Da) and a nanometer in size [1]. These characteristics allow them to diffuse throughout the body, binding promiscuously to both target and off-target sites. On the other hand, biologics encompass a wide range of biomolecular products ranging in size from short peptides to organs for transplantation [2–5]. As these products increase in size and complexity, so do their molecular and functional profiles.

Biotherapeutics are a subclass of biologics that are synthesized using recombinant DNA and/or hybridoma technologies. They include monoclonal antibodies, cytokines, growth factors, hormones, and other regulatory peptides [6]. They differ from small molecule drugs in that they are often hydrophilic and interact with a far more discrete set of targets making them an attractive alternative to traditional small molecule therapies associated with severe toxicities such as chemotherapeutics and immunosuppressants. For instance, antitumor antibodies can be conjugated to small molecule payloads, markedly decreasing the effective dose and toxic effects of the moiety. Once considered science fiction, the advancement of biologic technology has made treatments like enzyme replacement therapies, gene therapy, and tissue engineering a reality.

Biologics have some clear advantages over small molecule drugs, but they are not a panacea. Due to their high antigenic potential, biologics frequently stimulate an immunogenic response [7, 8]. Some clinical investigations of a biotherapeutic have reported antidrug antibody (ADA) formation incidence rates in >90% of patients [9–12]. These immunogenic responses sometimes result in altered efficacy and immunotoxicity profiles that can vary from patient to patient. Immunogenicity-related adverse events (AEs) may be as benign as a mild itchy rash or as serious as infusion reactions, anaphylaxis, and cytokine release syndrome (CRS) [13, 14]. As an example, tumor necrosis factor (TNF) inhibitors can activate autoantibodies leading to Drug-Induced Autoimmunity (DIA) [15]. Luckily, the majority of immunogenic responses are not toxic and do not lead to sustained autoimmune pathologies. Rather, encounters with ADAs affect efficacy by either accelerating or impeding clearance [16, 17]. It is in these instances that immunogenicity complicates the interplay of pharmacokinetics (PK) and pharmacodynamics (PD), which further obfuscates the development of a reliable preclinical model correlating with clinical events. Without these data, research and development (R&D) are severely impaired, further potentiating the already abysmal attrition rates (85.2%) of biologics once they reach phase 1 clinical trials [18]. As the library of biologics continues to grow, so does our need to understand how immunogenicity affects efficacy and safety. Here we set out to describe the biology of immunogenic response and its impact on the PK/PD of biologics. We do this in an effort to shed light on the challenges and pitfalls facing the successful development of safe and efficacious biologics.

## 2. Immunogenic Risk Factors and the Need for Predictive Tools

Attributes of both the biologic* and* the patient contribute to the risk of immunogenic response. The antigenicity of a therapy along with patient immune status can be used to predict the occurrence and severity of a potential immunogenic response. Patients with hyperreactive immune disease, autoimmunity, or history of biotherapeutic administration are all at an increased risk of reactions against biologics. Conversely, immunosuppressed patients are less likely to mount a response [19, 20]. Hypervigilant immune systems can be especially problematic when treating autoimmune diseases with biotherapeutics. For instance, an observational study monitored patients being treated for Crohn's disease with infliximab (IFX), a chimeric anti-TNF-alpha therapy [21]. It was found that patients with high anti-IFX antibodies in serum and low trough IFX levels correlated with lowered efficacy or poor IFX persistence. These clinical observations and immunoassays generated a PK profile of IFX that allowed clinicians to monitor Crohn's patients for bioanalytical markers that predicted treatment outcomes in the context of ADA. Nonetheless, this kind of data is often not applicable to biosimilars or other biotherapeutics and is difficult to validate across institutions administering identical therapies due to variances in sample collection,* in vitro* immunoassays, data analysis, and records. In order to standardize the clinical assessment of immunogenic response, procedures and standards must be applied and interpreted by predetermined criteria. The US Food and Drug Administration (FDA) and the European Medicine Agency (EMA) provide regulatory documents detailing such guidelines [22–24].

Genetically derived markers predicting immunogenicity could potentially be applied across all treatment groups and even across biotherapies. For instance, the Human Leukocyte Antigen (HLA) genes that encode for Major Histocompatibility Complex (MHC) class II haplotypes have been shown to play a role in the immunogenic response [25, 26]. A clinical study monitored patients diagnosed with Multiple Sclerosis (MS) treated with Interferon-Beta (INF-*β*). An association between a common MHC class II allele (DRB1^⁎^0701), present in 23% of the patients studied, and an increased likelihood of producing an ADA response against INF-*β* was discovered. Screening for this allele could guide future treatment plan decisions of MS patients. Establishing reliable and clinically relevant PK profiles of biotherapeutics and identifying genetic markers predictive of immunogenicity will allow clinicians to more effectively monitor and guide biotherapeutic administration.

### 2.1. Predicting Antigenicity

From discovery to clinic, immunogenicity must constantly be monitored, yet our current preclinical models have poor predictive power for clinically relevant immunogenicity [27]. For example, humanized therapies are designed to evade the immune system of patients, yet they are inherently antigenic in immunocompetent animal models and often exaggerate the antigenicity of the therapeutic agent. Nevertheless, the effort to combine preclinical data from* in silico*,* in vitro*, and* in vivo* methods remains worthwhile and may one day yield valid criteria for immunogenicity risk assessment.

*In silico* computational tools have broad applications in the identification and assessment of antigenicity. Some screen for leads such as cancer neoantigens and peptides for vaccine targets [28–30]. Others troubleshoot antigenicity by optimizing the design of peptide nanoparticles or by determining deimmunization methods for antileukemic L-asparaginase [31, 32]. As this technology matures and provides more reliable predictions, we will improve our ability to navigate the effects of immunogenicity.

*In vitro* modelling of antigenicity utilizes T cell assays, B cell assays, and mimotopes to identify potentially immunogenic epitopes [33, 34], whereas* in vitro* characterization of immunogenic response from serum samples includes qualitative and quasiquantitative immunoassays such as enzyme linked immunosorbent assays (ELISAs), radioimmunoassays (RIAs), and immunoradiometric assays (IRMAs) [35]. Cell-based assays remain a popular choice for modeling antigenicity via the immunophenotyping, proliferation tracking, and cytokine secretion profiling of immune cells [36, 37]. However, these techniques remain costly since they are labor and time intensive.* In vitro* modeling and* in vitro* characterization work together to describe mechanisms of immune activation as well as immunogenic markers/phenotypes belonging to both disease and nondisease states. Ideally, this information can be used to predict the magnitude and frequency of immunogenic response and to discern which patients are most at risk of developing this response. The limitations of the clinical utility of* in vitro* assays for immunogenic marker discovery were discussed in a previous section. However, predictive preclinical* in vitro* assays would help protect both patients and companies from the expense and risk associated with failed clinical trials. The development of such assays can be informed by selecting clinically tested therapeutic proteins and then validated if the clinically observed antibody response can be reconstructed in the preclinical setting [38]. Expanding the repertoire of valid yet simplified assays will push forward our preclinical evaluation of biologic design hopefully improving biologic design at its earliest stages.

Preclinical* in vivo* methods monitoring the antigenicity of a therapy have failed to correlate with clinical events and are considered to have low predictive value for immunogenic response in humans according to the International Conference on Harmonisation (ICH)* S6* guidance. However, they still hold value in the preclinical investigation of immunogenicity [39]. Nonhuman primates have proved successful in the modeling of toxicology, but an analysis of their use in nonclinical trials revealed that they are not particularly good for evaluating efficacy or immunogenicity since they both under-/overpredicted immunogenic response [40]. Consequently, researchers are still searching for ways to model human immunogenic response* in vivo*. One strategy is to engineer transgenic mice to express human antigens that tolerize them to humanized biotherapeutics. In these mice, researchers were able to model immune complex aggregation and extended-dose related ADA formation which closely mirror the phenomena observed in humans [41]. Vaccines are often evaluated in transgenic mice with a humanized haplotype, which has proved to be an effective tool for predicting CD4+ T cell activation [42, 43]. Savvy strategies like these are gradually improving our preclinical* in vivo* investigations, howbeit animal modeling remains an unreliable predictor of clinically relevant immunogenicity.

Over the past few decades, investigators have developed sophisticated assessment strategies and significantly expanded our understanding of immunogenicity. While regulatory agencies have agreed upon and established international guidelines for certain preclinical standards, the nonclinical evaluation of allergy and pseudoallergy remains unclear [44].

## 3. Immunotoxicity

Immunotoxicity is not unique to biologics. Penicillin is a classic example of a potentially lethal hypersensitivity reaction to a drug therapy, and DIA can be induced via small molecule therapies as well. As early as the 1950s, drug-induced lupus was documented in response to hydralazine [45]. Since biologics have greater immunogenic potential, they are more likely to elicit immunotoxic effects. Here we will briefly discuss immunogenic response, emphasizing ADA formation, and how these can affect the toxicity profile of the therapy.

Interestingly, many biotherapeutic agents are immunomodulators capable of stimulating or suppressing immune function. This immunogenicity is engineered to accomplish a therapeutic goal, whereas unwanted immunogenic response may impair therapeutic outcome by affecting safety and/or efficacy. Immunotoxicology is associated with four derangements of immune function: immunosuppression, immunostimulation, hypersensitivity, and autoimmunity [46, 47]. These derangements can result in an unintentional blockade of endogenous function, CRS, infusion reactions, and anaphylaxis. While these AEs can have serious consequences, the majority of immunogenic response elicits mild AEs or remains clinically silent. Prior to the start of a clinical trial, it is necessary that researchers evaluate the therapy in order to establish appropriate criteria for monitoring immunogenicity and to define unacceptable immunotoxicity that would halt/end the study.

Immunologically based AEs are often ADA-mediated. The formation of ADA is triggered when the classical pathway recognizes a “foreign” antigen via antigen presentation cells or by B/T cell mediated breaking of tolerance to humanized recombinant therapies [17, 48]. The latter functions via two pathways: the T cell dependent (TD) and the T cell independent (TI) pathway. TD accounts for 90% of ADA formation and relies on antigen stimulation of CD4+ T cell, which mounts a full adaptive immune response. TD activating therapeutic agents have an epitope that engages CD4+ T cells thus generating high affinity IgGs and memory B cells leading to a more persistent and robust response. In contrast, TI-ADA are thought to arise from the direct stimulation of B cell receptors (BCRs) by molecular therapies containing patterned structural motifs, that is, polymeric repeats or carbohydrate molecules, capable of crosslinking BCRs. TI activation generates pentavalent IgM or low-affinity IgG antibodies that bypass T cell mediated affinity maturation and isotype switching. This immature antibody response fails to induce memory phenotypes. T cell epitope screening should be considered along with the construct design of the therapy in order to mitigate ADA response by either selecting for immunosuppressive phenotypes like Tregs or avoiding stable epitopes all together.

Each biologic must be evaluated case by case for potential immunotoxicity. Careful attention is required when considering bispecific antibodies, antibody drug conjugates, and other products lacking an endogenous analog since they are more likely to elicit ADA formation [49]. For instance, Hemophilia A is a life-threatening genetic disease in which patients lack an essential clotting factor and is treated with Factor VIII replacement therapy [50]. Unfortunately this therapy triggers ADA response in up to a third of patients and often worsens the condition in patients with high ADA titers. Even though supplementary recombinant proteins are often constructed from the native protein sequences already expressed in patients they can elicit ADA which also neutralized the native proteins resulting in thrombocytopenia and red-cell aplasia [51, 52]. Additionally, tissue deposition of immune complexes can precipitate disease such as nephrotic syndrome and drug-induced vasculitis [53, 54]. Preclinical models and clinical precedence can provide clues pointing to possible immunogenic outcomes of a biologic in the clinic; however, immunotoxicity remains an unpredictable threat to patient safety.

## 4. The Impact of Immunogenicity on PK/PD and Efficacy

In order for a therapy to be successful once it reaches market, clinicians must have confidence in its therapeutic potential and be able to prescribe/administer the drug with a reliably effective dosing schedule. What the body does to the drug (PK) and what the drug does to the body (PD) need to be fully characterized and understood. This requires the accrual of data pertaining to treatment outcomes and clinical events that can be generalized to the biologic's target population. This has proven to be a challenging task for biotherapeutics, in part, because immunogenicity unpredictably perturbs PK/PD and efficacy. Regardless, the success of biologics would be improved by defining the impact of unwanted immunogenicity on PK profiles, especially since the primary reason for drug failure in phase II clinical trials is lack of clinical efficacy [55].

Small molecules and therapeutic proteins are comprised of entirely distinct molecular classes which are not only distinguished by their chemical profiles but their immunogenicity and PK profiles as well [17, 56]. Small molecule PK profiles can be interpreted from direct measurements of the active drug and its metabolites in serum; their ADME is well characterized and employs simplified compartment models. Their PD can be interpolated from relationships such as dose-exposure-response curves and does not require a nuanced understanding of the drug's mechanism of action. The PK/PD of biologics are driven by target-mediated drug disposition (TMDD) meaning that the high affinity of the therapy for its target (PD) strongly influences its PK. Theoretically, TMDD should yield a large volume of distribution with a high accumulation of therapy in its target tissue. However, tissue uptake can be slowed by a variety of mechanisms thus confining the therapy to its administration site. Currently, we do not understand ADME well enough to fully describe and characterize this interplay.

Serum measurements of biologics must consider the unbound, partially bound, and bound forms [57–59]. Distinguishing between the free and bound forms of the biologic and its metabolites is necessary in order to identify the effective fraction of bioactive drug in circulation. The terminal half-lives of these forms tend to vary, with the free form often displaying the shortest half-life [60]. Many of the well-established small molecule models employed by R&D fail to describe the PK/PD and ADME of biotherapeutics. Additionally, unwanted and unanticipated immunogenic response perturbs PK/PD thereby diminishing the predictive power of preclinical data. Our inability to describe and anticipate these phenomena hinders our ability to develop biologics that will prove efficacious and reliable once they reach clinical trial. Here we discuss what is known about immunogenicity's role in affecting PK/PD and how we hope to apply this knowledge in order to improve the predictive power of biologic models.

### 4.1. The Influence and Modes of Action of Immunogenicity on PK/PD Assessment

The immunogenic potential of biologics can be affected at any stage of their journey, starting with their route of administration or even after the biologic is cleared, by leaving a lasting impression on the immunomemory of the patient. Most biotherapeutics are large hydrophobic molecules that cannot survive the harsh conditions of the GI tract; therefore they have poor bioavailability by oral administration. Traditionally therapeutic proteins have been given intravenously (IV) maximizing bioavailability and minimizing immunogenic risk. However, this method is both inconvenient and expensive. Newer formulations allow for subcutaneous (SC), intraperitoneal, or inhalation routes [61–63]. SC administration is more cost effective and convenient than IV, yet it is often slow and incomplete. In particular for large molecules (>20 kDa) that tend to migrate more slowly through the extracellular matrix (ECM), SC poses an increased risk of ADA formation by dendritic cells processing which drain directly to the lymphatics. Depot formulations can aid absorption by including ECM degrading enzymes like hyaluronidase and by stabilizing proteins preventing degradation and aggregation [64]. Currently we are in need of formulations that can deliver an efficacious dose in affordable and convenient ways.

As was previously described, myriad patient and product-related factors can influence immunogenicity's effect on PK/PD and efficacy. ADAs are described by their mode of action, classifying them as either neutralizing ADAs (nADAs) or nonneutralizing ADAs (non-nADAs) [65]. The nADAs interact directly with biologics and/or bind to pharmacologically relevant sites, obscuring interactions between the therapy and its target, thereby decreasing the overall efficacy. Another classification categorizes ADA as sustaining or clearing, which indicates their influence on protein drug clearance [66]. Both nADA and non-nADA can either prolong or shorten the half-life of biologics. Most of the time immune complex formation, comprised of drug-ADA aggregates, will increase the clearance of the biotherapeutic. This phenomenon is most notable in patients with high affinity, high titer ADA. In some cases, isotype interactions with Fc receptors (FcR) also influence serum drug concentration. For IgGs, binding to the neonatal FcR (FcRn) increases bioavailability, possibly due to FcRn-mediated protection from catabolism at the injection site and/or draining lymphatics [67, 68]. Coupling immunoprotective technologies, such as Fc fusion proteins, with improved delivery formulations could stabilize circulating concentrations of biotherapeutics and their overall bioavailability. This effect will vary from patient to patient depending on factors like their MHC haplotypes and whether TD-ADA versus TI-ADA are triggered. Since we do not fully understand the patient and product-related factors governing this response, it is difficult to establish general guidelines defining an efficacious and reliable dosing regimen.

In order to characterize the effect of ADA on the PK profile of a biologic, an immunogenicity profile must be established. ADAs are a temporally evolving heterogeneous population circulating in serum alongside a multitude of endogenous proteins and antibodies [69, 70]. In most animals, ADAs become detectable within 2–4 weeks after the first dose administration and around 10 days in mice. They are usually polyclonal antibodies against multiple epitopes comprised of more than one isotype circulating at varying concentrations amongst a multitude of endogenous proteins. To overcome this daunting task of identifying and classifying ADA response, a standard bioanalytical schematic is employed: (1) sensitive screen, (2) confirmatory assay, and (3) functional characterization. The detection of these ADA is limited to capture antibody concentrations >20–1000 ng/mL with Kd of K-9 to K-8, respectively [71–73]. Occasionally the therapeutic is administered at such a low dose that it is rapidly consumed by the target site. In this absence of detectable circulating drug, PD measurements of target binding can correlate drug exposure. This method of analysis can also be useful in instances where a therapeutic protein has bioactive metabolites not measured by PK assays [74].

Studies looking at therapeutic antibodies, such as natalizumab and IFX, observed an increase in clearance and a reduction in efficacy when ADAs were persistent rather than transient throughout the first 12 weeks of therapy. They also found that clearing ADA formation was detectable 8–16 weeks after treatment initiation [8, 75, 76]. These labile kinetics suggest that* when* a measurement is taken significantly, it impacts the interpretation of the bioanalytics; therefore, dosing and sampling schedules should be well-defined and include peak and trough measurements as well as time points sampled long after the circulating drug has been cleared. Higher concentrations of ADAs are more likely to impact PK profiles and interfere with bioanalytical measurements making simultaneous quantification of both the biologic and ADA difficult. This hampers the ability to track the time course of ADA response. As a result, the impact of immune response on biologics cannot be fully investigated and our understanding remains incomplete.

Ultimately, the development of a physiologically based pharmacokinetic (PBPK) model akin to the small molecule compartment model that also accounts for immunogenicity would be ideal. Both “top down” and “bottom up” approaches are being used to derive mathematical models of ADME. For example, biodistribution coefficients (BC) can estimate tissue specific distribution of protein fragments based on molecular weight and plasma concentrations [77]. The BC50 values for most tissues were found to be ~35 kDa. Clinical derivation of these models could be achieved with a meta-analysis of clinical PK bioanalytical data, but inconsistent reporting of bioanalytical parameters regarding ADA response makes studies incomparable between institutions [78]. Fortunately, by collecting sparse or dense samples from many clinical studies, Population PK (PopPK) allows for the inclusion of data from a variety of un-/balanced designs. Most advantageously, PopPK looks at the target population of interest receiving clinically relevant doses rather than the healthy subjects in traditional PK studies. These studies enable the identification and measurement of variances and can point to potential explanations if identifying factors (demographic, environment, and pathophysiology) are found to correlate with altered PK. Currently, groups are working to validate parameters and refine models relating PK and immunogenicity; they have yet to design a reliable model generalizable to a diverse array of biotherapeutics.

ADAs affect both the clearance and efficacy of biologics. Building off of this basic tenant we have been able to partially classify the interplay between immunogenicity and PK/PD, yet we are unable to fully describe these phenomena. Unlike the PK/PD of small molecules, which began with bioanalytical measurements that later yielded theories to describe those measurements, the PK/PD of biologics is mostly understood through its apparent differences from small molecule ADME. We cannot rely on our current bioanalytical techniques to predict or describe PK/PD or clinical efficacy in the context immunogenic response.

## 5. Strategies for Overcoming the Pitfalls of Immunogenicity

As we have discussed, the PK/PD and the ADME of biologics can be dramatically affected by immunogenic response. So how then can we mitigate the consequences of those effects? We can approach this challenge from both R&D and clinical perspectives.

### 5.1. Biotherapeutic Production and Design

Design and manufacturing processes can mitigate or exacerbate the inherent antigenicity of biotherapeutics. Good manufacturing processes and quality assurance recognize the inherent heterogeneity of biologic batches. Antigenic impurities such as endotoxin contamination and detergents, aggregate formation, and variation in biologic activity must be kept to a minimum in order to ensure a safe and reliably efficacious product [79]. Careful handling of the protein product throughout the entire manufacturing process is critical in preventing aggregate formation. Not only are aggregated and misfolded proteins less efficacious, but also they precipitate immune complex formation and immunogenic response. Data suggest that proteins exposed to stress, freeze-thawing, and pH shifts with surface active agents or hydrophobic surfaces are more likely to form aggregates that can crosslink BCRs thereby inducing TI-ADA formation [80, 81]. Additionally, some biotherapeutics do not tolerate high concentration formulations well and may require more frequent dosing [82]. By improving production processes and conservatively administering these therapies, we can prevent the acquisition of these unnecessary antigenic properties.

Construct design is at the heart of biologic engineering. New and exciting therapies such as bispecific antibodies and antibody drug conjugates are expanding the utility and function of biotherapeutics, even so they may introduce antigenicity with their inadvertently novel structures. In fact, it was the drive to decrease antigenicity that evolved antibody therapies. Initial antibody therapies were murine-derived and found to be highly immunogenic. Eventually chimeric antibody technologies evolved until we achieved fully humanized antibodies. In theory, a humanized antibody, free of antigenic properties, should not be immunogenic. In reality, the immense variance of CD4+ T cell epitopes and MHC haplotypes allows for fully humanized antibodies to remain immunogenic in some patients [83, 84]. To mitigate this, deimmunization strategies can be used when designing protein therapeutics. This method utilizes* in silico* predictions of antigenic peptide sequences to identify potentially antigenic epitopes in a therapeutic protein. These peptides are then synthesized and their antigenicity is evaluated with* in vitro* binding assays to TCRs and MHC II receptors. Immunogenic epitopes are then mutated to a more tolerated sequence and subsequent constructs are iteratively screened. Tolerization is an ingenious twist on deimmunization that selects for and engineers biologics with Treg activating epitopes that can induce tolerance of the biologic [85]. Combining both deimmunization with tolerization has proved an effective method for reducing immunogenic response. Posttranslational modifications of biologics also appear to influence immunogenicity. For instance, glycosylation can shield antigens from ADA binding and retard processing. In line with this finding, a synthetic kind of glycosylation known as PEGylation can prevent protein aggregation and mask antigenic epitopes thereby reducing immune response [86]. However, sometimes this process introduces an immunogenic linker. Additionally, some forms of xeno-glycation may have immunostimulatory effects. The risk is especially high when eukaryotic organisms like yeast are used to express proteins, since they may introduce their own microorganism specific glycosylation patterns. For instance, yeast posttranslational processing adds mannose glycan which can bind to mannose receptors on human immune cells [87]. Though this is not an exhaustive list of all the possible considerations that should be taken into account when developing biologics, these examples illustrate the usefulness of optimizing a biologic's design to minimize immunogenicity.

### 5.2. The Clinical Approach

Clinicians already manage immunogenicity associated with a variety of pathologies and treatments such as rheumatoid arthritis and hematopoietic stem cell transplantation. Clinical strategies to mitigate immunogenic response include dose manipulation and immunosuppressive agents such as methrotrexate and prednisone. Some patients diagnosed with Infantile Pompe disease formed ADAs when treated with the enzyme replacement therapy, recombinant human acid *α*-glucosidase (rhGAA) [88, 89]. A subgroup of patients form nADAs, but additional work showed that this subgroup can be tolerized against rhGAA by complementing the replacement therapy with combinatorial immunosuppressive therapy comprised of rituximab and methotrexate. Fortunately, this tolerance persisted even after termination of B cell recovery and immunosuppression. Dosing-through is another method that has also proven to be a successful strategy in overcoming the deleterious effects of ADA on efficacy [90]. For MS patients receiving long term IFN-s therapy, nADAs have also proven to be problematic. Interestingly, recent studies have shown that high doses of IFN-sv32 result in lower nADA titers due to the saturation of these nADAs and the restoration of IFN-s binding to its receptor. High doses of IFN-s can also induce long term high dose tolerance of the immune system.

## 6. Summary and Conclusions

The advancement of biologics has revolutionized targeted therapy. By utilizing biologically derived tools we can administer drugs with complex and precise functions, which often mimic the body's natural processes. However, it is this key characteristic of molecular mimicry that potentiates the antigenicity of biologics and leads to undesirable ADA-mediated outcomes such as immunotoxicity and decreased efficacy. ADAs against biologics can alter ADME thereby greatly confounding the interpretation of PK/PD assessments. Thus, it is vital that we develop bioanalytical tools that can reliably predict and model the complex interplay between immunogenicity and PK/PD. Outside of patient immune status and exposure history, we do not currently possess adequate tools or knowledge of immunogenic markers to identify which patient-product interactions carry an increased risk of immunogenic response. Though we described various preclinical* in silico*,* in vitro*, and* in vivo* tools used for immunogenic risk assessment, these methods are not recognized as predictive. We must rely on clinical investigations. As novel biotherapeutic proteins continue to shape the course of modern medicine, we must work to fill these knowledge gaps in order to ensure that patients receive safe and effective treatments.
